# Facile Access to *Gleditsia microphylla* Galactomannan Hydrogel with Rapid Self-Repair Capacity and Multicyclic Water-Retaining Performance of Sandy Soil

**DOI:** 10.3390/polym14245430

**Published:** 2022-12-12

**Authors:** Chuanjie Liu, Meng Tang, Fenglun Zhang, Fuhou Lei, Pengfei Li, Kun Wang, Hongbo Zeng, Jianxin Jiang

**Affiliations:** 1 Engineering Research Center of Forestry Biomass Materials and Bioenergy, Ministry of Education, East China Woody Fragrance and Flavor Engineering Research Center of National Forestry and Grassland Administration, Beijing Forestry University, Beijing 100083, China; 2 Department of Chemical and Materials Engineering, University of Alberta, Edmonton, AB T6G 2V4, Canada; 3 Nanjing Institute for the Comprehensive Utilization of Wild Plants, Nanjing 211111, China; 4 Guangxi Key Laboratory of Chemistry and Engineering of Forest Products, College of Chemistry and Chemical Engineering, Guangxi University for Nationalities, Nanning 530006, China

**Keywords:** *Gleditsia microphylla* galactomannan, hydrogel, water retention, sandy soil, desertification

## Abstract

Sandy soil has poor water-holding performance, making it difficult for plants to survive, which worsens the deterioration of the ecological environment. Therefore, borax cross-linked *Gleditsia microphylla* galactomannan hydrogel (GMGH) was prepared, and its practicability as a water-retaining agent was analyzed. GMGH exhibited fast self-healing performance (150 s, ≈100%) and a high swelling index (88.70 g/g in pH 9). The feasibility of improving the water absorption and retention properties of sandy soil was explored by mixing different proportions (0.1, 0.3, 0.5 wt % sandy soil) of GMGH and sandy soil. The results showed that sandy soil had a more porous structure after adding 0.5 wt % GMGH, and its water absorption index increased from 15.68 to 38.12%. In an artificial climate box, the water-holding time of the sandy soil was extended from 3 to 23.5 days, and the cycles of water absorption and retention were more than 10 times. Therefore, GMGH has broad application prospects as a potential water-retaining agent for desertification control.

## 1. Introduction

Desertification has caused severe losses in agriculture, animal husbandry, and personal property in many countries and regions, gaining increased worldwide attention. The natural factors causing desertification are mostly drought, loose sandy sediments on the surface, and strong winds. The main anthropogenic factors are overgrazing [1,2], the unreasonable use of water resources [3,4,5,6], over-reclamation [7], river changes [8], and road construction [9]. Desertification leads to the destruction of land resources, reductions in available land area for agriculture and animal husbandry, degradation of the breeding capacity of the land, reduction in the number of plants, reduction in livestock-carrying capacity, and reduction in the yield per unit area of crops. Desertification makes it challenging to provide water and nutrients sustainably for the growth and development of plants; therefore, the priority of desertification control is to improve the water-holding capacity (WHC) of sandy soil to improve the quality of sandy soil. Providing reference solutions for desertification control has become an urgent problem.

Water is an integral part of plants that affects the biochemical reactions of enzymes, photosynthesis, and the metabolism of organic matter in plants [10,11]. Under high levels of sunlight, the atmospheric temperature increases, and the humidity correspondingly decreases. Meanwhile, the water content in plants decreases with an increase in transpiration rate, which accelerates the speed at which plants absorb water from the soil. Therefore, the water storage capacity of the soil directly affects the rooting, germination, leaf growth, flowering, and fruiting of plants. In addition, water can increase cell swelling pressure and maintain normal cell morphology [12], which is the reason why plants wilt during dry periods. Sandy soils have the disadvantages of low trace-element content, poor water-holding capacity, and considerable variations in soil temperature, which are not conducive to plant growth and reproduction [13]. Boron is an essential micronutrient for crop growth. Nadaf et al. [14] conducted a field experiment with a typical sandy loam soil that lacked available Zn and B. The application of zinc sulfate (alone) and borax (5 kg/ha) significantly increased the N, P, and K contents and their absorption in wheat straw and grain. Nadaf et al. also found that the application of Zn and B promoted the total absorption of Zn by peanuts [15]. Mandal et al. [16] observed a significant improvement in the nutrient absorption of mustard, following the mixed application of nitrogen, phosphorus, potassium fertilizer, borax, and zinc sulfate. In short, it is worthwhile exploring a strategy that can supplement the deficiency of boron in sandy soil and improve its water-holding capacity and pore structure.

Water-retaining agents, such as superabsorbent polymers (hydrogels), provide a strategy for sandy soil water conservation and ecological restoration [17]. A water-retaining agent is a type of functional material with a strong water absorption and retention capacity that can absorb water many times its weight. Water-retaining agents have been widely used in agriculture, forestry, and horticulture [18,19,20]. When a water-retaining agent is applied to the soil, a large amount of water forms a small, humid environment around plant roots. In the case of drought and water shortages, a water-retaining agent can provide water for plant growth and improve the plant’s survival rate. Jnanesha et al. [21] conducted a field study of red sandy soil in a semiarid environment in southern India. When 3000 g of Pusa-hydrogel was applied, the soil water-holding capacity improved significantly (5.55 kg/ha mm and 20.28%). Cechmankova et al. [22] used cellulose derivatives to cross-link citric acid to form hydrogels. Adding different levels of hydrogels to the soil had a positive effect on the water-holding capacity and basic properties of the soil. Agaba et al. [23] studied the addition of 0.2 and 0.4% polyacrylate hydrogels to soil to prolong the survival time of trees. Soil amendments can reduce soil hydraulic conductivity, plant transpiration, and soil evaporation. Saruchi et al. [24] used enzymes to synthesize *Scutellaria* gum and acrylic acid hydrogels, which improved the soil water-holding capacity (10% (*w*/*w*)), increased the water content of sandy loam and clay by 52 and 72%, respectively, and improved the WHC of the soil. Costa et al. [25] prepared hydrogels comprising potassium acrylate, acrylamide, and minerals. Hydrogels have high WHC and can reduce soil salinity, making them a potential conditioner for soil. Kareem et al. [26] synthesized slow-release fertilizer hydrogels using hydroxypropyl methylcellulose, polyvinyl alcohol, glycerin, and urea as raw materials. The water-retention capacity of the hydrogel in sandy soil showed a higher water-holding capacity than that in sandy soil without hydrogel. Womack et al. [27] used hydrogels to improve the pore network of soil, which can increase the retention of water and nutrients and also increase crop yield. Although these products can absorb a large amount of water, they may introduce secondary pollutants into the environment and have a high cost of use, which is not in line with the concepts of environmentally friendly and sustainable development.

Furthermore, research on the water-retaining agents created from natural polysaccharides is relatively limited. Natural polysaccharide materials are non-toxic, harmless, biocompatible, and biodegradable, and have a significant effect on ecological restoration [28,29,30,31]. In this study, *Gleditsia microphylla* gum (GMG), extracted from *Gleditsia microphylla* (GM) seeds, was selected as the raw material. GMG is mainly composed of galactomannan, which consists of linear (1→4)-linked β-D-mannopyranosyl and side chains of (1→6)-linked α-D-galactopyranosyl units with hydroxyl groups [32]. Borax cross-linked *Gleditsia microphylla* galactomannan hydrogel (GMGH) was obtained via a simple, easily accessible, environmentally friendly, and efficient method and its potential application as a water-retaining agent in sandy soil was analyzed.

## 2. Materials and Methods

### 2.1. Materials

*Gleditsia microphylla* gum (GMG, molecular weight 5.8 × 10^5^ Da, Mw/Mn = 1.591; mannose/galactose = 2.81) was a gift from Shanxi Yongyuan Biotechnology Co., Ltd. (Xi'an, China). Sodium tetraborate decahydrate (Borax, B_4_Na_2_O_7_·10H_2_O) was purchased from the Tianjin Chemical Reagent Factory (Tianjin, China). Methylene blue was purchased from Aladdin Bio-Chem Technology Co., Ltd. (Shanghai, China). All the chemical reagents used in this work were analytically pure and did not require additional purification. Sandy soil (particle size < 0.002 mm 9.05%, 0.02–0.002 mm 12.43%, 0.02–2 mm 78.52%) was dug from the shallow layer (0–30 cm) of the Ningxia Hui Autonomous Region (latitude and longitude: 38°24′ N, 105°97′ E) (Ningxia, China).

### 2.2. Synthesis of Hydrogels

**Scheme 1:**
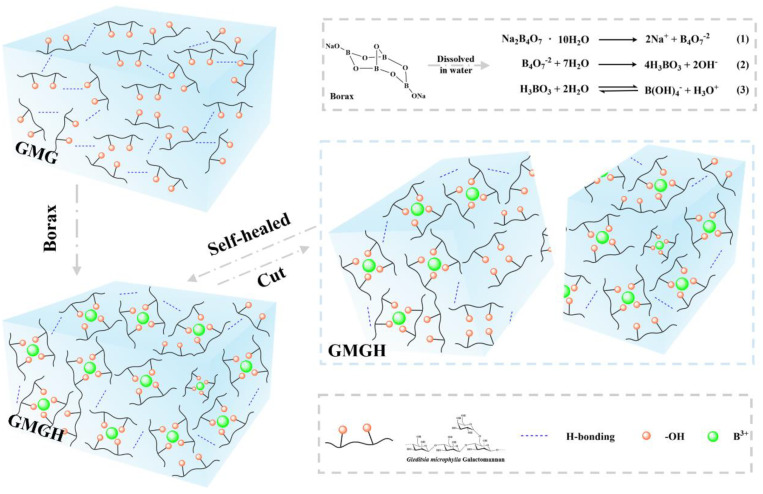
Formation mechanism of the GMGH.

First, GMG was extracted based on the method reported in the existing literature [32], then the GMG solution was prepared. GMG powder (0.5 g) was accurately weighed and put into a clean beaker. The GMG was dispersed evenly by adding 1 mL of absolute ethanol, and the total volume of the solution was fixed to 90 mL by adding deionized water. A uniform and stable solution of GMG was obtained after magnetic stirring at 30 °C for 1 h. Second, the hydrogel was prepared. According to the reaction mechanism (Scheme 1) and referring to previous research results [17,33], borax (15 wt % GMG) was dissolved in 10 mL of deionized water. After dissolution, the borax solution was added to the GMG solution by stirring at room temperature (RT) until a transparent and uniform borax cross-linked *Gleditsia microphylla* galactomannan hydrogel (GMGH) was formed. The blue *Gleditsia microphylla* galactomannan hydrogel (b-GMGH) was prepared using the same method, although methylene blue was added during the preparation process. Finally, freeze-dried and oven-dried GMGH (passing through 60-mesh) were prepared and used for the swelling properties tests, pore structure observations, sandy soil water retention experiments, and sandy slope simulation experiments.

### 2.3. Characterization of Hydrogels

Fourier-transform infrared spectroscopy (FT-IR, Model: ALPHA, Bruker Optics Ltd., Ettlingen, Germany) of GMG and GMGH was measured at wavelengths of 400–4000 cm^−1^ using the potassium bromide tableting method. X-ray diffraction (XRD, Model: D8 ADVANCE, Bruker Optics Ltd., Ettlingen, Germany) of the GMG and GMGH was performed in the 2θ range of 10–60°. The degree of crystallinity (DC) was calculated using the software provided by the manufacturer. Thermogravimetric analysis (TGA, Model: STA 449F5, NETZSCH-Gerätebau GmbH, Selb, Germany) of GMG and GMGH was conducted under nitrogen atmospheric conditions from 40 °C to 800 °C, with a fixed heating rate of 10 °C/min. Scanning electron microscopy (SEM, Model: S-3400N II, Hitachi Ltd., Tokyo, Japan) was used to observe the pore structure and arrangement of GMG and GMGH. Stereo zoom microscopy (SZM, Model: M205FA, Leica Microsystems Ltd., Wetzlar, Germany) was used to observe the morphology of GMGH, b-GMGH, self-healed GMGH, sandy soil, and soil–GMGH.

### 2.4. Self-Repair Performance

GMGH and b-GMGH were cut into hydrogel blocks of similar size. The hydrogels of different colors were quickly remolded using the mold, and the self-healing process of the hydrogel was achieved at room temperature without any assistance from an external force or stimulation. The process could also be discerned visually through the diffusion of methylene blue. Then, self-healed GMGH was placed in a cryogenic refrigerator at −80 °C for 24 h. Next, frozen GMGH was put into a vacuum dryer for freeze-drying for 48 h, and a self-repaired dried hydrogel was obtained. Freeze-dried GMGH was placed on a microscope platform to observe the network recovery of the incision. SZM confirmed the recovery of the self-repairing hydrogel network structure, and the swelling properties of hydrogels before and after self-repair were tested. The injection properties of the hydrogels were studied using a syringe.

### 2.5. Swelling Property of GMGH

According to previous studies, borax cross-linked galactomannan hydrogel becomes liquid when acid solutions are added [34]. Therefore, the swelling properties of the GMGH and self-repaired GMGH in aqueous solutions of 0.9% NaCl, tap water, and pH 9 ± 0.1 were studied. The weights of the GMGH and self-repaired GMGH were recorded after the complete absorption of the solution. The weight of hydrogels was recorded every five minutes until the weight remained unchanged, then Equation (1) was used to calculate the swelling index (SI):

SI = (W_1_ − W_0_)/W_0_ (1)

where W_0_ (g) is dried GMGH and W_1_ (g) is wet GMGH. All tests were repeated three times.

### 2.6. WHC of the Soil–GMGH

The WHC of the soil–GMGH was designed according to the methods reported in the existing literature [17] and slightly adjusted. First, the laboratory made a mold to obtain a sandy soil column with a height of 10 cm. Then, GMGH (0.1, 0.3, and 0.5 wt % sandy soil) was mixed with the sandy soil and transferred to abrasive tools. Next, the sandy soil and soil GMGH were saturated with tap water using a bottom-up method. The weight changes of the sandy soil and soil–GMGH were recorded at the turn of the day and night until the SI dropped to 10%. Through the climate incubator to simulate the ecological environment: for 8 h in the day, the temperature is 20 °C and the humidity is 60%, and for 16 h in the night, the temperature is 10 °C, and the humidity is 70%. The above process was continuously recycled more than ten times (each cycle was carried out according to the actual number of days), and the cyclic water retention of GMGH was investigated. Equation (2) was used to calculate the SI of the water absorption and retention of soil–GMGH. The dosages of GMGH (0.1, 0.3, and 0.5 wt % of sandy soil) were designated as GMGH-0.1%, GMGH-0.3%, and GMGH-0.5%, respectively.

SI = (W_3_ − W_0_ − W_2_)/(W_2_ + W_0_) (2)

where W_0_ (g) is dried GMGH, W_2_ (g) is dried sandy soil, and W_3_ (g) is wet soil–GMGH. All tests were repeated three times.

### 2.7. Sandy Slope Simulation Experiments

The sandy slope simulation experiment was conducted in the Ministry of Education Engineering Research Center of Forestry Biomass Materials and Bioenergy (Beijing, China). In this experiment, the slope angle was simulated and controlled using hanging hooks of different heights, which were set to 0°, 30°, 45°, 60°, and 90°. The preparation methods of the sandy soil and soil–GMGH were based on those described in Section 2.6. Morphological changes in sandy land with or without GMGH were observed by hanging hooks of different heights (hanging time 0.5 h). Then, the potential application value of the GMGH in sandy slope remediation and desertification control was identified.

## 3. Results and Discussions

### 3.1. Characterization of GMGH

**Figure 1:**
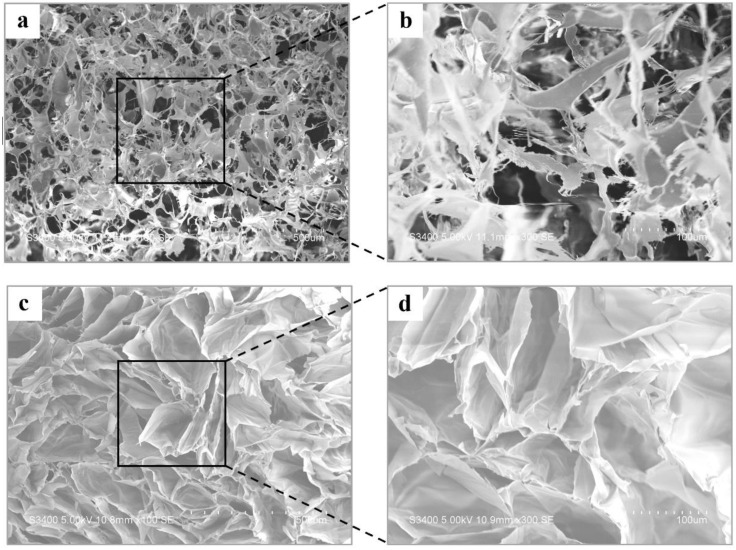
(**a**,**b**) Scanning electron microscopy (SEM) of 0.5 wt % GMG; (**c**,**d**) SEM of GMGH. The **a**,**c** scale bar = 500 μm, and the **b**,**d** scale bar = 100 μm.

Borax is an efficient crosslinker that can form conjugated acid-base pairs (B(OH)_4_^−^ and H_3_BO_3_) when dissolved in water [35]. The cross-linking mechanism (Scheme 1) of GMGH involves the complexation of borate ions (B(OH)_4_^−^) with the cis-ortho-hydroxyl groups of GMG to form a three-dimensional (3D) network structure. Based on the straightforward and green method of dynamic coordination cross-linking, dynamic reversible borate/di-diol bonds can quickly regulate the network structure of hydrogels, resulting in a domino effect (fast cross-linking) throughout the entire system. This complexation makes the internal structure of GMG disorderly (Figure 1a,b), interweaving into the hydrogel with a uniform and orderly pore structure (Figure 1c,d). The water-absorbing capacity and WHC of GMGH were enhanced because of the 3D network structure of the hydrogels. If the hydrogel is embedded in the sand layer to form a more porous structure in the sand-reinforced hydrogel, it can better promote root growth and water transport.

**Figure 2:**
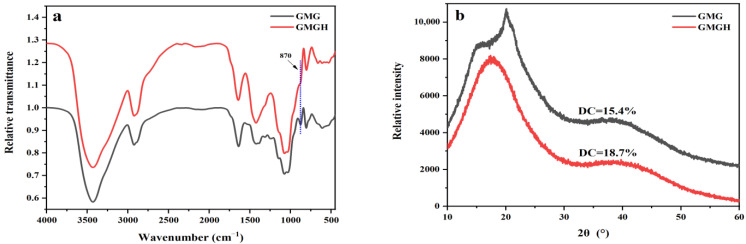

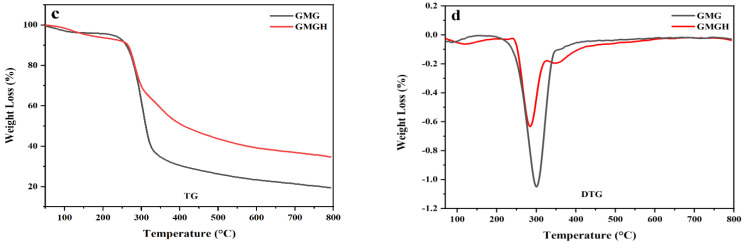
(**a**) FT-IR spectra of the GMG and GMGH powders; (**b**) X-ray diffraction patterns of GMG and GMGH; (**c**,**d**) thermogravimetric analysis of the GMG and GMGH powders.

From the FT-IR spectra shown in Figure 2a, it can be seen that GMGH has no new characteristic peaks compared with GMG. The broad peak at 3500–3400 cm^−1^ corresponds to the stretching vibration of the O-H group in GMG. The asymmetric stretching vibration absorption peak of CH_2_ appeared at 2920 cm^−1^, which was the characteristic absorption peak of GMG. The peak at 1630 cm^−1^ was formed by two O-H shears in the water molecules absorbed by GMG, while the peaks at 1150 and 1078 cm^−1^ were attributed to the C-O stretching vibration absorption peaks. The characteristic peak at 870 cm^−1^ was the stretching vibration peak of the GMG skeleton wherein the absorption intensity of GMG was weakened, indicating that the arrangement of the GMG network structure changed from a disorderly interwoven state to an orderly 3D network structure. The SEM images (Figure 1) also directly reflect this phenomenon. 

Figure 2b shows the XRD patterns of the GMG and GMGH powders, which indicate that GMG and GMGH exhibit a large dispersion peak. Crystallization is a type of ordered arrangement of molecular chains. Polymers with good symmetry can easily form ordered regions close to each other, and crystallinity represents the proportion of the crystalline regions. The DC of GMGH (18.7%) was higher than that of GMG (15.4%) because the disordered and intertwined polysaccharide skeleton reacted with borax to form a 3D network structure with a compact arrangement and a uniform pore structure (as shown in Figure 1).

The order of thermal stability was GMGH > GMG, as indicated by the TG curves in Figure 2c,d. The TG curves show two major mass loss regions of GMG and three major mass loss regions of GMGH. The first weight-loss region (50–105 °C) contained a small amount of free water in the sample. Next, the second weight-loss region (200–350 °C) of the GMG was the main weight-loss region (95–35%), which can be classified as polymer decomposition. For the GMGH, three mass-loss regions were observed. The first area (50–105 °C) was the initial water loss area, similar to GMG, whereas the second region (200–305 °C) was related to the degradation of the main chain of the GMGH, namely, the degradation of borate/di-diol between borax and cis-ortho-hydroxyl bonds in the hydrogel. The third zone (305–400 °C) can be classified as borax in the hydrogel system. The significant change in the TG and DTG analysis of GMGH is strong evidence of borate/di-diol cross-linking between the borax and cis-ortho-hydroxyl bonds. The results show that the dynamically reversible borate/di-diol bond has remarkable heat resistance. The heat-loss curve shows that borate/di-diol contributed to the heat resistance of GMGH, which is important for large-scale agroforestry applications and the industrial production of GMGH.

### 3.2. Self-Healing and Injection Ability

**Figure 3:**
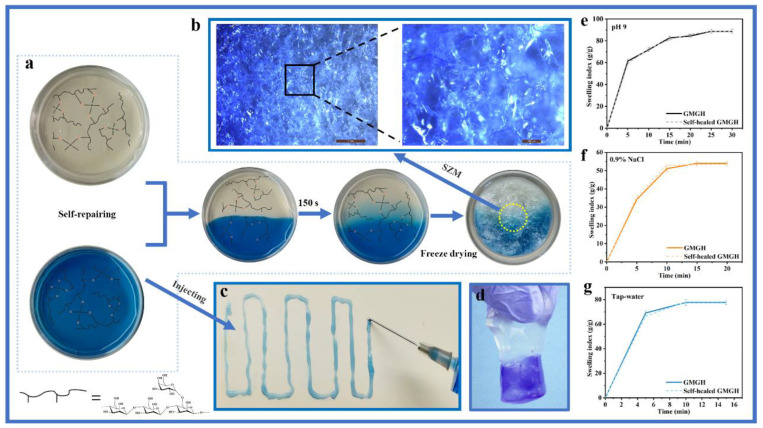
(**a**,**d**) Photos showing the self-healing behavior of GMGH; (**b**) image of the network pore structure of freeze-dried self-repaired GMGH; (**c**) GMGH can be injected using needles; (**e**) swelling index in water at pH 9; (**f**) Swelling index in 0.9% NaCl solution; (**g**) Swelling index in tap water.

It can be seen intuitively that the 3D network structure of the hydrogel was re-established, as shown in Figure 3a,b,d, as was corroborated by the diffusion of the color. This shows that the dynamic reversible borate/di-diol bonds can be rapidly reconnected to achieve recovery of the hydrogel network structure. As shown in Figure 3c, GMGH was easily extruded via a 5 mL injector, which was expected to be applied in the field of biomedicine. Injectable performance can be important not only as a carrier for drug delivery but also as a carrier for slope spraying or aerial seeding. Therefore, there is no need to worry about the operating of deep plowing and weeding destroying the structure of the hydrogel network because it can be recovered quickly. The dynamic reversible borate/di-diol formed via the side O-H groups of GMG and the B(OH)_4_^−^ groups endows GMGH with excellent self-repairing properties and injectability.

### 3.3. Water Absorption and Retention of GMGH

The water absorption and retention in 0.9% NaCl, tap water, and a pH of 9 are shown in Figure 3e–g. In a relatively short period of time (5–30 min), the hydrogel reached swelling equilibrium, showing that the largest and fastest swelling was at pH 9, and the smallest swelling was in 0.9% NaCl solution. Under alkaline conditions, this is probably due to the expansion of the cross-linked chain formed by increased hydrogen bonding [20,36]. That is, when the pH value increases, the equilibrium moves toward the formation of boric acid ions; the OH- from borate hydrolysis is one of the basic conditions required for cross-linking. In saline solutions, owing to the high osmotic pressure around the hydrogel, GMGH absorption capacity was significantly reduced, which reduced the osmotic pressure between the inner and outer phases and prevented the solvent water from freely entering the GMGH network [37]. The highest expansion indices of GMGH in the 0.9% NaCl, tap water, and pH 9 solutions were 54.00, 77.72, and 88.70 g/g, respectively.

The self-healing capability of GMGH was indirectly reflected by investigating the SI of self-repaired GMGH. Similarly, freeze-dried self-repaired hydrogels (Figure 3) were soaked in different solutions to observe their SI. Figure 3e–g show that the swelling property of the self-repaired GMGH was not significantly different from that of the original hydrogel. It can be concluded from the SI that the 3D network structure of GMGH was completely restored.

### 3.4. Determination of WHC of Sandy Soil

**Figure 4:**
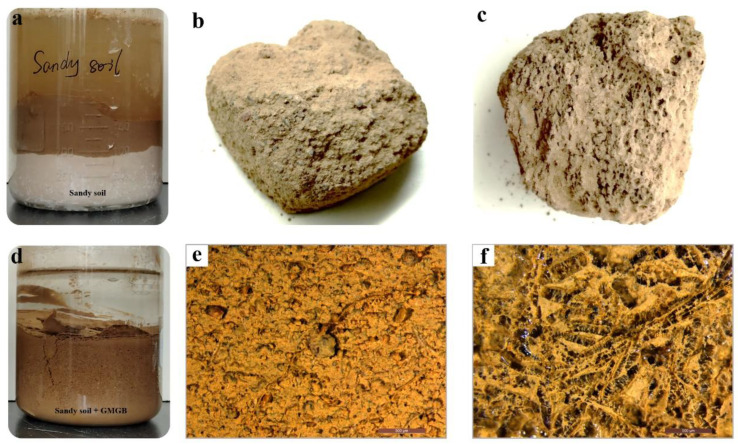
(**a**) The sandy soil absorbs water slowly; (**b**) dried sandy soil; (**c**) dried sandy soil–GMGH; (**d**) the sandy soil with GMGB absorbs water quickly; (**e**) enlarged SZM image of the sandy soil; (**f**) enlarged SZM image of the sandy soil–GMGH.

Soil moisture affects the physical and chemical properties and fertility of the soil and is a critical factor in determining plant growth [38]. Improving the soil WHC can improve soil drought resistance and increase the agricultural yield and income of farmers. The ability of the soil to absorb water is known as the water-holding capacity, which mainly depends on the capillary force in the soil pores. Sandy soil has low porosity, poor water retention, and hardens after water loss, as shown in Figure 4b,e. However, sandy soil with GMGH can absorb and retain water quickly (Figure 4a,d, Video S1 in the Supplementary Materials). After natural drying, the soil–GMGH maintained good structural porosity, which was significantly improved, as shown in Figure 4e,f. Intuitively speaking, the pore structure was conducive to the growth of plant roots and the flow of water.

**Figure 5:**
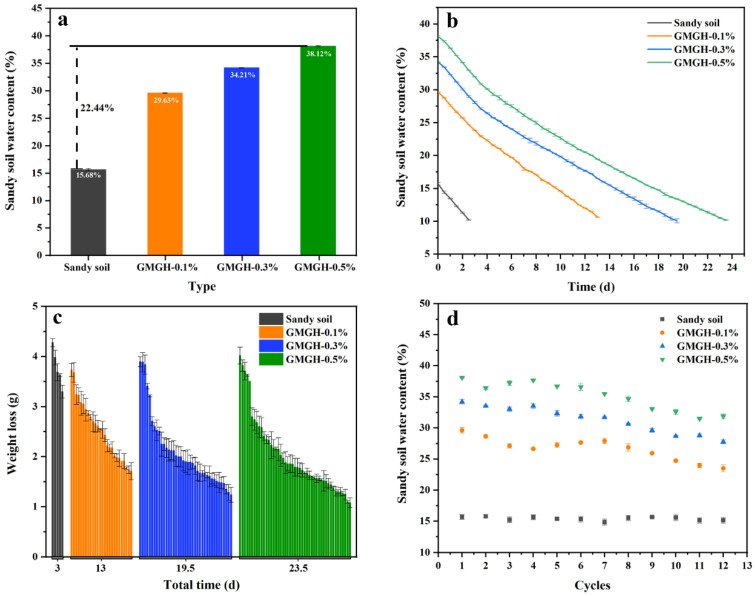
(**a**) The maximum SI of the sandy soil and soil–GMGH in tap water; (**b**) the relationship between the SI and time of soil–GMGH in the artificial climate box; (**c**) weight changes in the soil and soil–GMGH in each period (12 h); (**d**) repeated water absorption and the water retention index of soil and soil–GMGH.

Soil–GMGH not only held water quickly but also had remarkable stability in terms of water loss and circulation, as shown in Figure 5. GMGH effectively improved the soil water retention at low doses (0.1, 0.3, and 0.5 wt % sandy soil). The results show that the maximum WHC of sandy soil without any treatment was 15.68%; that is, 100 g of dried soil held 15.68 g of tap water before reaching saturation. When 0.1 wt % GMGH particles were mixed with sandy soil, the WHC of the sandy soil increased by 13.95%, from 15.68 to 29.63%. Adding 0.3 wt % of GMGH particles improved the WHC of sandy soil from 15.68 to 34.21%, an increase of 18.53%. The WHC of the sandy soil increased by 22.44% from 15.68 to 38.12% when 0.5 wt % of GMGH powder was added. The addition of GMGH significantly improved the WHC of sandy soil, making it a potential water-retaining agent for improving sandy soil and effectively controlling soil erosion.

Sufficient water is a vital condition that must be met to ensure plant survival. If there is a lack of water, the plants wither. Then, fertile land begins to degrade, and the ecological environment further deteriorates. In this part of the experiment, the effect of GMGH as a water-retaining agent on the WHC of sandy soil was investigated, including the times of water loss, absorption, and retention. According to previous studies, it is difficult to maintain normal plant activities when the soil moisture content is less than 10% [39,40]. Therefore, the water absorption and retention indexes of the sandy soil decreased to 10% at the end of the experiment, and the time that was taken was recorded.

Figure 5 shows that the different amounts of GMGH (0.1, 0.3, and 0.5 wt % sandy soil) were evenly distributed in the sandy soil, saturated by irrigating with tap water. There is no doubt that soil-GMGH-0.5% had the largest expansion index and the longest water-holding time in the artificial climate chamber (parameters: 8 h in the daytime, temperature 20 °C, humidity 60%, and 16 h at night, temperature 10 °C, humidity 70%). Specifically, the sandy soil took 3 days for the SI to decrease from 15.68 to 10%. GMGH-0.1% took 13 days for the SI to decrease from 29.63 to 10%, which was 4.33 times that of the sandy soil. GMGH-0.3% took 19.5 days for the SI to decrease from 34.21 to 10%, which was 6.5 times that of the sandy soil. GMGH-0.5% took 19.5 days for the SI to decrease from 38.12 to 10%. Therefore, the maximum water-holding time of GMGH-0.5% was 7.83 times that of the sandy soil; that is, GMGH increased the sandy soil water-holding time (SI decrease to 10%) from 3 to 23.5 days.

Figure 5c shows the weight change of the sandy soil in each period (day or night) until the expansion index was reduced to 10% in the artificial climate chamber. The water loss of the sandy soil, GMGH-0.1%, GMGH-0.3%, and GMGH-0.5% in each period showed a downward trend, from 4.28 to 3.3 g, 3.73 to 1.71 g, 3.91 to 1.24 g, and 4.02 to 1.08 g, respectively. GMGH can overcome the poor water storage capacity limitations of sandy soil and provide sustainable water for plant growth.

### 3.5. Cyclic Water Absorption and Water Retention Capacity of the Soil-GMGH

The stability of a water-retaining agent is its ability to absorb water multiple times and maintain a relatively stable SI. This directly determines the planting cost for farmers and reflects labor productivity. Figure 5d shows the cyclic water retention of the sandy soil and soil-GMGH in the artificial climate box (parameters: 8 h in the day, the temperature was 20 °C, the humidity was 60%, and 16 h at night, the temperature was 10 °C, and the humidity was 70%). To make the experiment closer to reality, sandy soil was plowed to further investigate the stability of the GMGH after each cycle of water absorption and loss. It could still maintain a relatively stable water storage capacity after 12 water absorption and retention cycles of soil-GMGH. Deep tillage did not affect the water retention performance of soil-GMGH, which indicates that soil-GMGH also has self-healing performance and can promote rapid recovery of the soil-GMGH pore network. Above all, borate/di-diol bonds not only exhibit dynamic reversibility but also exhibit stable recovery in complex sandy soil environments. Thus, GMGH is potentially an excellent water-retaining agent.

### 3.6. Results of the Sandy Slope Simulation Experiment

**Figure 6:**
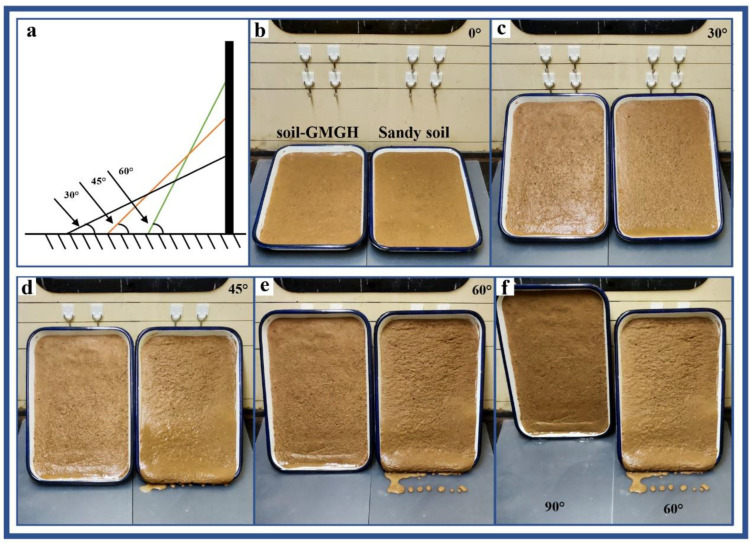
Sandy slope simulation diagram. (**a**) Cross-section angle; (**b**) wet soil–GMGH and sandy soil at 0°; (**c**) wet soil–GMGH and sandy soil at 30°; (**d**) wet soil–GMGH and sandy soil at 45°; (**e**) wet soil–GMGH and sandy soil: 60°; (**f**) wet soil–GMGH and sandy soil at 90° and 60°.

Slopes and gullies are the landscape features that are most prone to soil erosion; that is, the environment is destroyed under the action of a variety of external forces, which results in the loss of water and soil resources. Soil erosion can be reduced or avoided through the construction of scientific protective engineering measures, which is of great significance in the conservation of the ecological environment. In this study, the feasibility of water retention on sandy slopes was explored by simulating sandy slopes with different angles. In addition, the simulation diagram was shown in Figure 6. Pallets (30 cm long, 21 cm wide, and 3 cm high) were used as simulated grinding tools for sloped land. The relative inclination angles between the different slopes can be seen directly in Figure 6a.

Figure 6b–f shows the slope of the sandy soil (the right side of the photograph) and soil GMGH (the left side of the photograph) with tap water. Notably, the soil–GMGH has a stable grasping ability. When the slope increases from 0 to 45°, the primary forms of the sandy soil and soil-GMGH remain unchanged. When the tray angle increases to 60°, sandy soil without GMGH creates an apparent landslide, i.e., the sediment begins to slide, while the soil–GMGH can still maintain a relatively stable state. Surprisingly, when the tray of soil–GMGH rises to 90° (Figure 6f), it can remain stable without any sliding. Therefore, as a potential water-retaining agent, GMGH can not only significantly improve the water absorption and retention capacity of the sandy soil but also play the role of a windbreak and ensure sand fixation on sandy slopes, which offers potential ecological restoration material.

## 4. Conclusions

In this study, a simple and convenient method to prepare a water-retention agent based on *Gleditsia microphylla* galactomannan was developed. GMGH, when combined with sandy soil, can increase the WHC of sandy soil by more than 22% and absorb water circularly (more than 10 times). The newly formed 3D network pore structure of sandy soil is conducive to crop growth, yielding certain economic benefits. This study provides a strategy for desertification control that has particular reference significance and practical value.

## Data Availability

Data will be made available on request.

## Supplementary Materials

The following supporting information can be downloaded at: https://www.mdpi.com/article/10.3390/polym14245430/s1.

## Abbreviations

GM: *Gleditsia microphylla*; GMG, *Gleditsia microphylla* gum; GMGH, *Gleditsia microphylla* galactomannan hydrogel; SI, swelling index; WHC, water-holding capacity; FT-IR, Fourier transform infrared spectroscopy; TGA, thermogravimetric analysis; XRD, X-ray diffraction; SEM, scanning electron microscopy; SZM, stereo zoom microscopy.
